# A benchmark for dose-finding studies with unknown ordering

**DOI:** 10.1093/biostatistics/kxaa054

**Published:** 2021-01-04

**Authors:** Pavel Mozgunov, Xavier Paoletti, Thomas Jaki

**Affiliations:** Department of Mathematics and Statistics, Lancaster University, Lancaster, UK; Université Versailles St Quentin & INSERM U900 STAMPM, Institut Curie, Paris, France; Department of Mathematics and Statistics, Lancaster University, Lancaster, UK and MRC Biostatistics Unit, University of Cambridge, Cambridge, UK

**Keywords:** Benchmark, Combination trial, Dose finding, Partial ordering, Power likelihood

## Abstract

An important tool to evaluate the performance of a dose-finding design is the nonparametric optimal benchmark that provides an upper bound on the performance of a design under a given scenario. A fundamental assumption of the benchmark is that the investigator can arrange doses in a monotonically increasing toxicity order. While the benchmark can be still applied to combination studies in which not all dose combinations can be ordered, it does not account for the uncertainty in the ordering. In this article, we propose a generalization of the benchmark that accounts for this uncertainty and, as a result, provides a sharper upper bound on the performance. The benchmark assesses how probable the occurrence of each ordering is, given the complete information about each patient. The proposed approach can be applied to trials with an arbitrary number of endpoints with discrete or continuous distributions. We illustrate the utility of the benchmark using recently proposed dose-finding designs for Phase I combination trials with a binary toxicity endpoint and Phase I/II combination trials with binary toxicity and continuous efficacy endpoints.

## 1 Introduction

There has been growing interest in combination dose-finding trials of several agents administered simultaneously. Whilst coadministration can induce improved activity, designing such trials is more challenging compared to single-agent ones. Many single-agent dose-finding designs are based on the assumption that toxicity increases monotonically with the dose. However, in a combination study, there are combinations that cannot be ordered with respect to increasing toxicity. As a result, many novel model-based (see reviews by Riviere and others, 2015; Hirakawa and others, 2015, and references therein) and curve-free methods (e.g. Mozgunov and Jaki, 2019, 2020) were proposed to relax this assumption. Similarly to single-agent designs, the performance of these methods is conventionally assessed by simulation studies. These studies use combination-toxicity relationships, scenarios, which are chosen by the researchers themselves. This adds subjectivity to the assessment as the performance depends on the chosen scenario. The problem of selecting the scenarios is of relevance in dose-finding trials generally. To reduce the subjectivity, O’Quigley and others (2002) proposed an evaluation tool, the *nonparametric optimal benchmark*, that provides a scenario-specific evaluation of the performance in terms of the proportion of correct selections (PCS) of single-agent designs. When no strong prior information is used, the benchmark provides the highest PCS *a design* can achieve under the given simulation scenario. Occasionally, dose-finding methods can result in PCS that exceeds the PCS provided by the benchmark under certain scenarios. This is known as super-efficiency (Paoletti and others, 2004) and might be an indication of the design favoring particular doses (either due to the prior information or design specification) which the benchmark can reveal.

The benchmark was proposed under the assumption of monotonically increasing toxicity which typically holds in single-agent trials. Whilst the original benchmark can be also applied to dose-finding studies with unknown orderings (Mozgunov and others, 2020), the obtained upper bound for the PCS is expected to be less sharp compared to the setting when the monotonicity assumption holds.

To illustrate this, consider a hypothetical setting of a dual-agent combination study without early stopping. Assume that there are three increasing doses of agent *A* denoted by *a*_1_, *a*_2_, *a*_3_ and three increasing doses of agent *B* denoted by *b*_1_, *b*_2_, *b*_3_. Denote the combination of two doses *a_k_* and *b_l_* by *d_kl_* where the first index refers to the *k*th dose of agent *A*, and the second index refers to the *l*th dose of agent *B*. There are nine drug combinations in the trial. Assume that the toxicity of combinations increases within each agent. This corresponds to at least one of the subscripts in *d_kl_* increasing. However, some of the combinations cannot be ordered, for example, it is unknown whether *d*_12_ is more or less toxic than *d*_31_ as the dose of *A* is increased while the dose of *B* is decreased. Due to this uncertainty, there are 42 *complete* orderings of these combinations (see Supplementary materials available at *Biostatistics* online) that satisfy the monotonicity assumption within each agent. We will call the orderings satisfying this assumption the *feasible* orderings. The term “complete” refers to the feasible orderings of *all* nine combinations with respect to increased toxicity. The term “partial ordering” will refer to an ordered subset of combinations that could be arranged in increasing toxicity order (Wages and others, 2011a).

Consider a binary toxicity endpoint—occurrence of dose-limiting toxicity (DLT). The toxicity of *d_kl_* is characterized by toxicity probability *p_kl_*, *k*, *l* = 1, 2, 3. Suppose that the objective is to find the combination with the toxicity probability closest to 30% and assume a sample size of *n* = 36 patients. We would like to evaluate a design under the two scenarios given in Table 1.

The distance between the probabilities closest to the target is nearly the same under both scenarios, although the locations of the target combinations are different. Under Scenario 1, the target combinations are *d*_12_ and *d*_21_. Following the monotonicity assumption, one of these combinations must be in the *second* position in any feasible complete ordering. Under Scenario 2, there are more possibilities of the location of the target combinations. Combination *d*_13_ can be in the *third, fourth, fifth*, *sixth*, or *seventh* positions of the complete orderings, while *d*_22_ can be in the *fourth, fifth,* and *sixth* positions (Table 6 in Supplementary material available at *Biostatistics* online). Therefore, one can expect that it is more challenging to find the target combinations under Scenario 2. However, the original benchmark (implemented by Wages and Varhegyi, 2017) disregards the uncertainty in the ordering and treats these scenarios similarly providing nearly the same PCS (Table 1).

In this article, we propose an extension of the benchmark for studies with unknown ordering. The novel benchmark accounts for both the uncertainty in the target combination locations within each feasible ordering and distribution of these orderings. We show that, compared to the original benchmark, the proposal can provide a sharper bound on the performance of dose-finding designs relaxing monotonicity assumption while capturing the whole distribution of selections. In contrast to the recent benchmark proposal for dual-agent combination dose-finding trials by Guo and Liu (2018), the novel approach uses the original concept of complete information by O’Quigley and others (2002), which assumes that outcomes of each patient can be observed at all combinations. The benchmark, therefore, uses all available information about each patient, while accounting for the fact that combinations that cannot be ordered carry limited information about each other.

In line with extensions of the original benchmark to categorical and continuous endpoints (Cheung, 2014; Mozgunov and others, 2020), the proposal allows for an arbitrary number of endpoints having either discrete or continuous distributions. We demonstrate how the novel benchmark can be applied to a Phase I/II dual-agent combination study evaluating a binary toxicity endpoint and a Phase I/II combination study with binary toxicity and continuous efficacy endpoints.

The rest of the manuscript proceeds as follows. We review the benchmark by O’Quigley and others (2002) in Section 2. The construction of the benchmark for partial ordering in the combination setting with a single binary endpoint is given in Section 3 and extended to trials with multiple endpoints in Section 4. Section 5 demonstrates applications of the proposed benchmark before we conclude with a discussion.

## 2 The Benchmark for Single-Agent Studies With Binary Endpoint

Consider a Phase I clinical trial with a binary toxicity outcome, DLT or no DLT, *n* patients and *M* increasing doses of a drug, *c*_1_, … , *c_M_*. Let $Y_{j}^{\left(i\right)}$ be a Bernoulli random variable taking value $y_{j}^{\left(i\right)}=0$ if patient *i* has experienced no DLT at dose *c_j_* and $y_{j}^{\left(i\right)}=1$ otherwise. This distribution of $y_{j}^{\left(i\right)}$ is characterized by probability *p_j_* such that $p_{j}=\mathbb{P}\left(Y_{j}^{\left(i\right)}=1\right)$ for *j* = 1, … , *m* and any *i*. The goal of the trial is to find the maximum tolerated dose (MTD) defined as the dose having the probability of toxicity closest to the target level, γ, typically between 20% and 35%.

The benchmark uses the concept of *complete information*. For a given patient, the complete information consists of the vector of outcomes (DLT or no DLT) at all doses (in contrast to an actual trial, in which patients can only be assigned to one) assuming that *p*_1_, … , *p_m_* are known. Formally, the information about the DLT of patient *i* at each dose is summarized in a single value, *u*^(*i*)^ ∈ (0, 1), which is drawn from a uniform distribution, *𝒰* (0, 1), and is known as a toxicity profile of patient *i*. The variable *u*^(*i*)^ is transformed to response $Y_{j}^{\left(i\right)}=0$ for doses with *p_j_* < *u*^(*i*)^ and to $y_{j}^{\left(i\right)}=1$ otherwise. The procedure is repeated for *n* patients, which results in the vector of responses for each dose level $y_{j}=\left(y_{j}^{\left(1\right)},\ldots ,y_{j}^{\left(n\right)}\right),$ *j* = 1, …, *M*. Note that the procedure is not sequential—responses for previous patients are not required to compute the complete information for the next ones. Therefore, there is no assignment criterion used by the benchmark. Let *R*(*y_j_*, *γ*) be a summary statistic for the dose *c_j_*, upon which the decision about the MTD selection is based. For example, in many Phase I trials with binary outcomes, $R\left(y_{j},\gamma \right)=\left|\frac{\sum_{i=1}^{n}y_{j}^{\left(t\right)}}{n}-\gamma \right|$ is a conventional choice. Therefore, *c_j_*, for which *R*(*y_j_*, *γ*) is minimized among all *j* = 1, …, *M*, is declared as the MTD in a single trial. The procedure is repeated for *Z* simulated trials. For each dose, the proportion of simulated trials that choose this dose as the MTD is computed. This proportion is the benchmark’s estimate for the upper bounds of the PCS. Importantly, the benchmark is an evaluation tool and is not obtainable in actual trials. It can however be used at the planning stage to evaluate the performance of a dose-finding design.

## 3 Benchmark for Phase I Combination Studies with Binary Endpoint

### 3.1 Setting

Using the notations above, consider a Phase I dual-agent trial with *a*_1_, …, *a_K_* doses of drug *A*, *b*_1_, …, *b_L_* doses of drug *B*, their combinations *d_kl_*, and a binary toxicity outcome, DLT or no DLT. Similarly to the single-agent setting, let $Y_{kl}^{\left(i\right)}$ be a Bernoulli random variable taking value $y_{kl}^{\left(i\right)}=0$ if patient *i* has experienced no DLT at combination *d_kl_* and $y_{kl}^{\left(i\right)}=1$ otherwise, *k* = 1, …, *K*, *l* = 1, …, *L*. The distributions of $y_{kl}^{\left(i\right)}$ are characterized by probabilities $p_{kl}=\mathbb{P}\left(Y_{kl}^{\left(i\right)}=1|d_{kl}\right)$ that increase with the dose of each compound. The goal is to find the maximum tolerated combination (MTC), the combination corresponding to a risk of toxicity closest to the target value γ . We use the following example throughout this section to demonstrate the novel benchmark construction.

Example Consider the simplest dual-agent trial with 2 doses of drugs *A* and *B* with *a*_1_ < *a*_2_ and *b*_1_ < *b*_2_, and four combinations, *d*_11_, *d*_12_, *d*_21_, *d*_22_, and suppose the target toxicity is 20%. There are two *complete* orderings satisfying the monotonicity assumption within each agent

(3.1) $$(a)d_{11}\to d_{21}\to d_{12}\to d_{22}\;\text{and}\;\;\;(b)d_{11}\to d_{12}\to d_{21}\to d_{22}.$$

Then, the *partial* orderings are *d*_11_ → *d*_12_ → *d*_22_ and *d*_11_ → *d*_21_ → *d*_22_.

To provide an upper bound for the PCS, the benchmark for unknown ordering proposed in this work answers two questions: (i) “What is the probability of finding the true MTC if the ordering is known?” and (ii) “What is the probability of an ordering being identified as a correct one?.” The original bench-mark answers the first question only, and hence, provides a less accurate PCS upper bound. The general construction of our proposal is outlined below.

- Assuming the true ordering is known, obtain the patients’ responses at each combination;
- Fixing these responses but not using the information about the true ordering, compute the probability that these responses were obtained from a given ordering;
- Under the given ordering, find the combination selected and assign the corresponding probability of this ordering being identified as a correct one to this combination selection.

### 3.2 Construction: generating responses

To address the first question, we start by following the original benchmark. Assume that *p_kl_* is known for all *k*, *l*. We will refer to these probabilities as the *true scenario*. In the simulation setting, this sequence is known.

As before, assume that the toxicity profile of patient *i* is summarized in a single value *u*^(*i*)^ ~ 𝒰(0, 1) meaning that patient *i* can tolerate combinations *d_kl_* with *p_kl_* < *u*^(*i*)^ and would experience a DLT if given combinations *d_k′l′_* associated with *p_k′l′_* > *u*^(*i*)^. Then, the patient’s response can be written as $y_{kl}^{\left(i\right)}=1$ for *p_kl_* > *u*^(*i*)^ and as $y_{kl}^{\left(i\right)}=0,$ otherwise. Assume that there is a sample of *n* patients with tolerances *u*(1), …, *u*^(*n*)^ and denote the number of DLTs for these *n* patients at each combination by $x_{kl}=\sum_{i=1}^{n}y_{kl}^{\left(i\right)},$ *k* = 1, …, *K*, *l* = 1, …, *L*. Estimates of the probabilities of toxicity at *d_kl_* can then be found as ${\hat{p}}_{kl}=\frac{x_{kl}}{n}.$ Note that the patient outcomes are generated using the true scenario and, hence, a true ordering.

Example (Continued) Assume that the true probabilities of toxicity *p_kl_* for *d*_11_, *d*_12_, *d*_21_, *d*_22_ are given by *p*_11_ = .10, *p*_12_ = .30, *p*_21_ = .20, *p*_22_ = .40.

$$\left[\begin{array}{ll} 0.10 & 0.30\\ 0.20 & 0.40 \end{array}\right]$$

implying that the ordering (*a*) in Equation (3.1) is correct. Assume that *n* = 10 patients with toxicity profiles *u*^(1)^ = 0.59, *u*^(2)^ = 0.01, *u*^(3)^ = 0.29, *u*^(4)^ = 0.28, *u*^(5)^ = 0.81, *u*^(6)^ = 0.26, *u*^(7)^ = 0.72, *u*^(8)^ = 0.31, *u*^(9)^ = 0.95, *u*^(10)^ = 0.11 were generated. This corresponds to the following numbers of DLTs at each combination *x*_11_ = 1, *x*_12_ = 5, *x*_21_ = 2, *x*_22_ = 6.

We now fix the number of DLTs obtained and find how likely is that they were drawn from each of the feasible orderings.

### 3.3 Construction: identifying the probability of each ordering

Fixing the values of the true toxicity probabilities and the number of DLTs at each combination, consider now *S* complete feasible orderings for these values. We assume that the values of toxicity probabilities are known but we do not know which probability goes with which combination. Denote the probability of DLT given *d_kl_* under ordering *s* by $q_{kl}^{\left(s\right)},$ and let s* be a correct ordering. Consequently, $q_{kl}^{\left(s^{⋆}\right)}=p_{kl}$ for all *k*, *l*. Probabilities $q_{kl}^{\left(s\right)}$ are constructed as all possible permutations (with respect to the complete feasible orderings) of the true probabilities *p_kl_*.

Example (Continued) There are two feasible orderings in the considered example, *S* = 2. Consequently, $q_{kl}^{\left(s\right)},$ *s* = 1, 2 are

$$q^{\left(1\right)}=\left[\begin{array}{ll} 0.10 & \underline{0.30}\\ \underline{0.20} & 0.40 \end{array}\right],\;q^{\left(2\right)}=\left[\begin{array}{ll} 0.10 & \underline{0.20}\\ \underline{0.30} & 0.40 \end{array}\right],$$

where the values corresponding to the uncertainty in the monotonic ordering are underlined. The second question to be answered by the benchmark can be reformulated as “How likely it is that the sequence of $q_{kl}^{\left(s\right)}$ (also referred to as ordering *s*) is a correct one, given the observed responses *x_kl_*?.” Using the data generated for all *n* patients, the proposed benchmark computes

(3.2) $$\mathbb{P}\left(P_{kl}=q_{kl}^{\left(s\right)}|x_{kl}\right),s=1,\ldots ,s$$

for all *d_kl_*. Note that the probability of toxicity, *P_kl_*, is now considered as a random variable itself, which can take a discrete number of values which are defined by the true toxicity probabilities that are feasible at the position (*a_k_*, *b_l_*).

Using Bayes Theorem, the probability (3.2) is proportional to the likelihood of observing *X_kl_* given the DLT probability $P_{kl}=q_{kl}^{\left(s\right)},$ which equals

$$\mathbb{P}\left(X_{kl}=x_{kl}|P_{kl}=q_{kl}^{\left(s\right)}\right)=\text{Bin}\left(x_{kl},n,q_{kl}^{\left(s\right)}\right)=\left(\begin{array}{c} n\\ x_{kl} \end{array}\right)q_{kl}^{\left(s\right)}x_{kl}{\left(1-q_{kl}^{\left(s\right)}\right)}^{n-x_{kl}},$$

where Bin (·) is the density function of the binomial random variable. Let *t_kl_* be the number of values *P_kl_* can take, and let $h_{kl}^{\left(s\right)}=\mathbb{P}\left(P_{kl}=q_{kl}^{\left(s\right)}\right)$ be a prior probability that the toxicity probability at *d_kl_* under ordering *s* is $q_{kl}^{\left(s\right)}$ such that $\sum_{s=1}^{s}h_{kl}^{\left(s\right)}=1.$ If all feasible values corresponding to combination *d_kl_* are a priori equally likely then $h_{kl}^{\left(s\right)}=\frac{1}{t_{kl}}.$ Then, the posterior probability that the DLTs at *d_kl_* were obtained from the probability $q_{kl}^{\left(s\right)}$ given DLTs *x_kl_* is proportional to

(3.3) $$\mathbb{P}\left(P_{kl}=q_{kl}^{\left(s\right)}|x_{kl}\right)\propto \left(\begin{array}{c} n\\ x_{kl} \end{array}\right)q_{kl}^{{\left(s\right)}^{x_{kl}}}{\left(1-q_{kl}^{\left(s\right)}\right)}^{n-x_{kl}}\times h_{kl}^{\left(s\right)}.$$

Using these posterior probabilities for each combination corresponding to some ordering *s*′, we find the probability of this ordering to be identified as a correct one. We allow for different importances of the contributions of various combinations to the posterior probability of the responses to be obtained from ordering *s*′. Specifically, we assume that it is proportional to

(3.4) $$\mathbb{P}\left(s=s^{\prime }|x_{11},\ldots ,x_{KL}\right)\propto \prod_{k,l}{\left[\left(\begin{array}{c} n\\ x_{kl} \end{array}\right)q_{kl}^{\left(s^{\prime }\right)x_{kl}}{\left(1-q_{kl}^{\left(s^{\prime }\right)}\right)}^{n-x_{kl}}\times h_{kl}^{\left(s^{\prime }\right)}\right]}^{w_{kl}},$$

where *w_kl_* is a weighting parameter corresponding to combination *d_kl_*. The RHS in (3.4) is the power likelihood with parameter *w_kl_* > 0 used in Bayesian analysis to control the learning rate of Bayesian update (Holmes and Walker, 2017). Values 0 < *w_kl_* < 1 give less prominence to the data than the Bayesian model. In the context of the study with uncertainty in monotonic ordering, the weights *w_kl_* represent different contributions the combinations provide about the probability of complete ordering. Intuitively, one learns about the combinations within the same partial ordering more than about combinations that cannot be ordered. Then, the probability of ordering *s*′ can be written as

(3.5) $$\mathbb{P}\left(s=s^{\prime }|x_{11},\ldots ,x_{KL}\right)=\frac{\prod_{k,l}{\left[\text{Bin}\left(x_{kl},n,q_{kl}^{\left(s^{\prime }\right)}\right)\times h_{kl}^{\left(s^{\prime }\right)}\right]}^{w_{kl}}}{\sum_{s=1}^{s}\prod_{k,l}{\left[\text{Bin}\left(x_{kl},n,q_{kl}^{\left(s\right)}\right)\times h_{kl}^{\left(s\right)}\right]}^{w_{kl}}}.$$

Below, we consider the following form of the weight function

(3.6) $$w_{kl}={\left(1+\text{\#}\left\{\text{ combinations that cannot be ordered wrt }d_{kl}\right\}\right)}^{-1}$$

corresponding to a higher weight if one has less uncertainty about the toxicity probability of combination *d_kl_* with respect to other combinations. Note that the form of the weight function above is an arbitrary choice and other forms of the weight function that resembles the idea of assigning less weight to combinations carrying less information can be used.

Example (Continued) Under 1, 5, 2, 6 DLTs observed at combinations *d*_11_, *d*_12_, *d*_21_, *d*_22_, the probabilities of observing these responses *x_kl_* under *q*^(1)^ and *q*^(2)^ in *n* = 10 patients are

$$\begin{array}{ll} \text{Bin}\left(x_{11}=1,P_{11}=q_{11}^{\left(1\right)}=q_{11}^{\left(2\right)}=0.10\right)\approx 0.39, & \text{Bin}\left(x_{22}=6,P_{22}=q_{22}^{\left(1\right)}=q_{22}^{\left(2\right)}=0.40\right)\approx 0.11,\\ \;\;\;\;\text{Bin}\left(x_{12}=5,P_{12}=q_{12}^{\left(1\right)}=0.30\right)\approx 0.10, & \text{Bin}\left(x_{12}=5,P_{12}=q_{12}^{\left(2\right)}=0.20\right)\approx 0.03,\\ \;\;\;\;\;\text{Bin}\left(x_{21}=2,P_{21}=q_{21}^{\left(1\right)}=0.20\right)\approx 0.30, & \text{Bin}\left(x_{21}=2,P_{21}=q_{21}^{\left(2\right)}=0.30\right)\approx 0.23. \end{array}$$

The weight values for each combinations (3.6) are equal to *w*_11_ = *w*_22_ = 1 and $w_{12}=w_{21}=\frac{1}{2}.$ The weight *w*_11_ represents that the responses at *d*_11_ and *d*_22_ provides information for all four combinations, while the responses *d*_12_ and *d*_21_ do not provide information about each other. Assume that a priori any of the probability values specified in the true scenario at the anti-diagonal elements of the combination-toxicity matrix are equally likely, $h_{12}^{\left(1\right)}=h_{12}^{\left(2\right)}=h_{21}^{\left(1\right)}=h_{21}^{\left(2\right)}=\frac{1}{2}.$ Then, the probabilities of each ordering can be found as 𝕡(*s* = 1|·) 0.69 and 𝕡(*s* = 2|·) = 0.31.

Note that the posterior probabilities of the orderings in Equation (3.5) should not be used to select a single correct ordering to base further inference on. Instead, these probabilities will define each ordering’s contribution to the selection probabilities obtained by the novel benchmark.

### 3.4 Construction: computing the proportion of selections under the benchmark

Once the probability of each ordering *s* = 1, …, *S* is found, the benchmark proceeds as follows. Fix the ordering *s*′ and find the estimates of the toxicity probabilities at combination *d_kl_*, ${\hat{q}}_{kl}^{\left(s^{\prime }\right)}$ under this ordering using the toxicity profiles *u*^(1)^, …, *u*^(*n*)^ generated before and computed as ${\hat{q}}_{kl}^{\left(s^{\prime }\right)}=\frac{\sum_{i=1}^{n}I\left(u^{\left(t\right)}<q_{kl}^{\left(s^{\prime }\right)}\right)}{n}.$ Under ordering *s*′, the MTC is selected using

(3.7) $$R\left({\hat{q}}_{kl}^{\left(s^{\prime }\right)},\gamma \right)=\left|{\hat{q}}_{kl}^{\left(s^{\prime }\right)}-\gamma \right|.$$

The combination which minimizes criterion (3.7), is selected with the probability that the ordering *s*′ is selected, *P*(*s* = *s*′|·). Using the same toxicity profiles, the procedure is repeated for all *S* orderings. The resulting estimates are the probability of selection of each combination.

Example (Continued) If ordering *s* = 1 is selected, then the estimates of the toxicity probabilities are ${\hat{q}}_{11}^{\left(1\right)}=0.10,$ ${\hat{q}}_{12}^{\left(1\right)}=0.50,$ ${\hat{q}}_{21}^{\left(1\right)}=0.20,$ ${\hat{q}}_{22}^{\left(1\right)}=0.60.$ Targeting the toxicity probability of 20%, the combination *d*_21_ is selected using criterion (3.7). As ordering *s* = 1 is selected with probability 0.69, then *d*_12_ is also selected with probability 0.69. Similarly, if the ordering *s* = 2 is selected, then the estimates are ${\hat{q}}_{11}^{\left(1\right)}=0.10,$ ${\hat{q}}_{12}^{\left(1\right)}=0.20,$ ${\hat{q}}_{21}^{\left(1\right)}=0.50,$ ${\hat{q}}_{22}^{\left(1\right)}=0.60,$ and *d*_12_ is selected with probability 0.31. Therefore, the probability of combinations *d*_11_, *d*_12_, *d*_21_, *d*_22_ being selected in this simulated trial with the observed DLTs are (0.00, 0.31, 0.69, 0.00), respectively.

Finally, by generating *Z* simulated trials (each with *n* new toxicity profiles), the probability of each combination selection can be found in every simulated trial; the mean probability over *Z* simulations will be the benchmark’s estimate of the combinations’ selections. These proportions of each combinations’ selections are used to obtain the proportion of *correct* selections (PCS) for a given definition of a “correct” combination set by the clinicians in a trial.

A step-by-step guide on how the benchmark for studies with unknown ordering and a binary endpoint can be constructed based on *Z* simulated trials is given in Algorithm 1.

Algorithm 1 **Computing a partial ordering benchmark for a single binary outcome**

1. Specify *S* feasible complete orderings and toxicity probabilities *p_kl_* for all combinations, *k* = 1 …, *K*, *l* = 1 …, *L*.

2. Generate a sequence of patients’ profiles ${\left\{u_{i}\right\}}_{i=1}^{n}$ from *𝒰*(0, 1), transform *u_i_* to $y_{kl}^{\left(i\right)}=1$ if *p_kl_* > *u_i_* and store $x_{kl}=\sum_{i=1}^{n}y_{kl}^{\left(i\right)},$ *k* = 1 …, *K*, *l* = 1, *L* 𝕏 = [*x*_11_, …, *x_KL_*].

3. Compute the probability of ordering *s*′ being selected, 𝕡(*s* = *s*′|𝕏), *s*′ = 1, …, *S*.

4. For each ordering *s*′, *s*′ = 1, …, *S*, compute estimates ${\hat{q}}_{kl}^{\left(s^{\prime }\right)},$ the criterion $R\left({\hat{q}}_{kl}^{\left(s^{\prime }\right)},\gamma \right),$ and find the target combination *d*_k*l*_ under ordering *s*′ and set *Q*_k*l*_ (*z*) = 𝕡(*s* = *s*′|𝕏) .

5. Repeat steps 2–4 for *z* = 1, …, *Z* simulated trials.

6. Use ${\hat{Q}}_{kl}=\sum_{z=1}^{Z}Q_{kl}(z)/Z$ as the selection proportion of *d_kl_*, *k* = 1 …,*K*, *l* = 1 …, *L*.

An application of the proposed benchmark to evaluate a dose-finding design for a Phase I dual-agent combination study is provided in Section 5.1.

## 4 Benchmark for Combination Studies with Multiple Endpoints

We now extend the proposed benchmark to accommodate a growing number of combination studies evaluating more than a single toxicity endpoint. For example, there are several novel designs for Phase I/II combination studies evaluating binary toxicity and binary or continuous efficacy simultaneously (Hirakawa, 2012; Wages and others, 2014; Yuan and others, 2016). For this, we build on the benchmark for continuous endpoints (Mozgunov and others, 2020).

Consider a Phase I/II trial with toxicity outcome $T_{kl}^{\left(i\right)}$ and efficacy outcome $E_{kl}^{\left(i\right)}$ with Cumulative Density Functions (CDFs) *F*_*t*,*kl*_ and *F*_*e*,*kl*_, respectively, at *d_kl_* for patient *i*. Assume that *F*_*t*,*kl*_ and *F*_*e*,*kl*_ are parametrized by *θ*_*t*,*kl*_ and *θ*_*e*,*kl*_, respectively, and *f*_*t*,*kl*_(·), *f*_*e*,*kl*_(·) are the corresponding density functions.

For patient *i*, the toxicity profile is given by $u_{t}^{\left(i\right)}\in (0,1)$ and the efficacy profile is given by $u_{e}^{\left(i\right)}\in (0,1).$ Then, following Mozgunov and others (2020), the toxicity and efficacy responses, $t_{kl}^{\left(i\right)}$ and $e_{kl}^{\left(i\right)},$ patient *i* would have at combination *d_kl_* can be found as $t_{kl}^{\left(i\right)}=F_{t,kl}^{-1}\left(u_{t}^{\left(i\right)}\right),$ and $e_{kl}^{\left(i\right)}=F_{e,kl}^{-1}\left(u_{e}^{\left(i\right)}\right).$ Repeating the procedure for *n* patients, one can obtain the vectors $t_{kl}=\left(t_{kl}^{\left(1\right)},\ldots ,t_{kl}^{\left(n\right)}\right),$ $e_{kl}=\left(e_{kl}^{\left(1\right)},\ldots ,e_{kl}^{\left(n\right)}\right)$ for each *d_kl_*.

Fixing the *values* of the toxicity and efficacy parameters, *θ*_*t*,*kl*_, *θ*_*e*,*kl*_, and the toxicity and efficacy responses *t*_kl_, *e_kl_*, consider now, *S_t_* orderings of the values of *θ*_*t*,*kl*_, and *S_e_* orderings of the values of *θ*_*e*,*kl*_. We assume that the values of parameters *θ*_*t*,*kl*_, *θ*_*e*,*kl*_ are known, but similar to the setting above, we do not know which parameters go with which combination. For example, in the setting with binary toxicity and efficacy responses, these parameters are probabilities of toxicity and efficacy, respectively. Denote the toxicity parameter associated with combination *d_kl_* under ordering *s_t_* by $\lambda_{t,kl}^{\left(s_{t}\right)},$ the efficacy parameter associated with combination *d_kl_* under ordering *s_e_* by $\lambda_{e,kl}^{\left(s_{e}\right)},$ and let $s_{t}^{⋆},$ $s_{e}^{⋆}$ be the true orderings (true scenario). Consequently, $\lambda_{t,kl}^{\left(s_{t}^{*}\right)}=\theta_{t,kl}$ and $\lambda_{e,kl}^{\left(s_{e}^{*}\right)}=\theta_{e,kl}$ for all *k*, *l*. As before, parameters $\lambda_{t,kl}^{\left(s_{t}\right)},$ $\lambda_{e,kl}^{\left(s_{e}\right)}$ are constructed as all possible permutations of the true parameter values *θ*_*t*,*kl*_, *θ*_*e*,*kl*_ with respect to complete feasible orderings, respectively.

Again, in the setting of the benchmark, one would like to answer the question “What is the probability of identifying correct orderings $s_{t}^{⋆}$ and $s_{e}^{⋆}$ among all feasible orderings given the responses *t_kl_*, *e_kl_*, *k* = 1, …, *K*, and *l* = 1, …, *L*?.” The probability of ordering $S_{t}=S_{t}^{'}$ being identified as a correct one is

(4.8) $$\mathbb{P}\left(s_{t}=s_{t}^{\prime }|t_{11},\ldots ,t_{KL}\right)=\frac{\prod_{k,l}{\left[ℒ\left(t_{kl},\lambda_{t,kl}^{\left(s_{t}^{\prime }\right)}\right)\times h_{t,kl}^{\left(s_{t}\right)}\right]}^{w_{kl}}}{\sum_{s_{t}=1}^{S_{t}}\prod_{k,l}{\left[ℒ\left(t_{kl},\lambda_{t,tl}^{(stl}\right)\times h_{t,kl}^{(s_{t}t}\right]}^{w_{kl}}},$$

where 𝓛 is the likelihood function $ℒ\left(t_{kl},\lambda_{t,kl}^{\left(s_{t}^{\prime }\right)}\right)=\prod_{i=1}^{n}f\left(t_{kl}^{\left(i\right)},\lambda_{t,kl}^{\left(s_{t}^{\prime }\right)}\right)$ and $h_{t,kl}^{\left(s_{t}^{\prime }\right)}$ is the prior probability that *θ*_*t*,*kl*_ equals $\lambda_{t,kl}^{\left(s_{t}^{\prime }\right)}$ under *s*′. Similarly, one can find $\mathbb{P}\left(s_{e}=s_{e}^{\prime }|e_{11},\ldots ,e_{KL}\right).$ Then, the probability of identifying orderings $s_{t}^{\prime }$ and $s_{e}^{\prime }$ simultaneously, is

$$\mathbb{P}\left(s_{t}=s_{t}^{\prime },s_{e}=s_{e}^{\prime }|\cdot \right)=\frac{\mathbb{P}\left(s_{t}=s_{t}^{\prime }|\cdot \right)\times \mathbb{P}\left(s_{e}=s_{e}^{\prime }|\cdot \right)}{\sum_{u,v}^{s_{t},S_{e}}\mathbb{P}\left(s_{t}=u|\cdot \right)\times \mathbb{P}\left(s_{e}=v|\cdot \right)}.$$

Note that the weights *w_kl_* have the same interpretation as above, and the function in the form given in Equation (3.6) is studied further.

Under each combination of orderings *s_t_* and *s_e_*, using previously generated responses *t_kl_*, *e_kl_*, one can find the target combination (TC) that optimizes some decision criterion *R*(·). Then, this combination is selected with probability 𝕡(*s_t_*, *s_e_*|·). The procedure repeats for *Z* simulated trials. Algorithm 2 provides step-by-step guidance on how the benchmark for studies with partial ordering and *Q* endpoints with discrete or continuous distributions can be constructed.

Algorithm 2 **Computing a partial ordering benchmark for studies with several endpoints**

1. Specify CDFs *F*_*q*,*kl*_ for *q* = 1, …, *Q* endpoints and all combinations *k* = 1 …, *K*, *l* = 1 …, *L*. Specify *S*_1_, …, *S_Q_* orderings for each endpoints, and criterion *R*(·).

2. Generate profiles $U_{q}^{\left(i\right)}$ for all patients *i* = 1, …, *n* and all endpoints *q* = 1, …, *Q*.

3. Apply the quantile transformation $y_{q,kl}^{\left(i\right)}=F_{q,kl}^{-1}\left(u_{q}^{\left(i\right)}\right)$ for *i* = 1, …, *n*, *q* = 1, …, *Q*, *k* = 1, …, *K* and *l* = 1, …, *L*, and store *y*_*q*,*kl*_.

4. Compute the probability (4.8) of ordering *s_q_* = 1, …, *S_q_* being a correct one, *q* = 1, …, *Q*.

5. For each combination of orderings $\left(s_{1}^{\prime },\ldots ,s_{Q}^{\prime }\right),$ compute the values of the criterion *T*(·), find the target combination *d*_k*l*_ and set $Q_{k^{⋆}l^{⋆}}(z)=\mathbb{P}\left(s_{1}=s_{1}^{\prime },\ldots ,s_{Q}=s_{O}^{\prime }|\cdot \right).$

6. Repeat steps 2–5 for *z* = 1, …, *Z* simulated trials.

6. Use ${\hat{Q}}_{kl}=\sum_{z=1}^{z}Q_{kl}(z)/Z$ as the selection proportion of *d_kl_* for *k* = 1 …,*K*, *l* = 1 …, *L*.

Note that the construction of the benchmark above concerns a general case of an arbitrary (and possibly different) number of orderings of toxicities and efficacies. However, there are cases in which it might be reasonable to assume that the order of toxicities is the same as the order of efficacies, *s_t_* = *s_e_* = *s*. Then, the construction of the probabilities of orderings for a pair of endpoints reduces to the computation of the probability of orderings for a single endpoint but using both toxicity and efficacy data. Specifically, in case of the toxicity and efficacy orderings being the same, probability (4.8) can be found as

$$\mathbb{P}\left(s=s^{\prime }|\cdot \right)=\frac{\prod_{k,l}{\left[ℒ\left(t_{kl},\lambda_{t,kl}^{\left(s^{\prime }\right)}\right)\times ℒ\left(e_{kl},\lambda_{e,kl}^{\left(s^{\prime }\right)}\right)\times h_{kl}^{\left(s\right)}\right]}^{w_{kl}}}{\sum_{s=1}^{s}\prod_{k,l}{\left[ℒ\left(t_{kl},\lambda_{t,kl}^{\left(s\right)}\right)\times ℒ\left(e_{kl},\lambda_{e,kl}^{\left(s\right)}\right)\times h_{kl}^{\left(s\right)}\right]}^{w_{kl}}},$$

where $h_{t,kl}^{\left(s_{t}\right)}=h_{e,kl}^{\left(s_{e}\right)}=h_{kl}^{\left(s\right)}.$ Applications of the proposed benchmark to evaluate a Phase I/II dual-agent design for binary toxicity and continuous efficacy when toxicity and efficacy orderings can differ is provided in Section 5.2, and an evaluation in the setting of binary toxicity and efficacy endpoints with coinciding orderings is given in Supplementary material available at *Biostatistics* online.

## 5 Examples

Below, we provide two examples of how the novel benchmark can be used at the planning stage of a trial to provide a more accurate evaluation of a design to be used in the study. Specifically, we consider a Phase I combination clinical trial with a binary toxicity endpoint, and a Phase I/II clinical trial with binary toxicity and continuous efficacy endpoints.

### 5.1 Evaluation of dose-finding designs for combination studies with binary toxicity

The original benchmark for single-agent trials was found to provide an accurate upper bound for the model-based design, continual reassessment method (O’Quigley and others, 2002, CRM). Therefore, it is of interest to evaluate how the extension of CRM relaxing the monotonicity assumption proposed by Wages and others (2011a), Partial Ordering CRM (POCRM), performs compared to the novel benchmark for partial ordering. Additionally, we also evaluate the Bayesian I2D design by Wang and Ivanova (2005).

#### 5.1.1 Setting

Consider a dual-agent combination study with three doses of drug *A* and five doses of drug *B* (resulting in fifteen combinations), *n* = 60 patients, and a binary toxicity endpoint. The goal of the trial is to identify the MTC corresponding to the target probability of toxicity *γ* = 0.30. We consider ten combination-toxicity scenarios (Table 2) considered by Riviere and others (2015) in their review of dose-finding designs for combination studies

On top of the true probabilities of toxicity, one needs to specify the feasible toxicity orderings to apply the proposed benchmark. The total number of orderings satisfying the monotonicity assumption within each agent is *S* = 6006 (see Supplementary material available at *Biostatistics* online for procedures computing the orderings), and we assume that all orderings are equally likely prior to the trial. Finally, in line with the objective function of the dose-finding designs under evaluation, we consider absolute distance decision criterion (3.7) for the dose selection.

We evaluate the maximum likelihood (two-stage) version of the POCRM design proposed by Wages and others (2011b) and the I2D design by Wang and Ivanova (2005). The core idea of the POCRM is to run several CRM models under different orderings and allocate patients sequentially based on the most likely ordering. The maximum likelihood POCRM requires a sequence of initial patients’ allocations to be used until at least one DLT and one non-DLT have been observed. After this, the combination selection will be governed by the POCRM. The initial escalation phase as proposed by Wages (2015) is considered. Furthermore, the POCRM requires the specification of a set of orderings that will be tried by the design. We consider six orderings as proposed by Wages and Conaway (2013); Wages (2015) that were found to lead to good operational characteristics. The two-stage design is implemented in R-package pocrm (Wages and Varhegyi, 2013). We also evaluate the I2D design as specified by Riviere and others (2015).

We also include the benchmark proposed by Guo and Liu (2018) for trials with a single binary endpoint. We refer to this benchmark as “GL.” It is based on the critical information introduced by the authors that is argued to offer a middle ground between the complete information and data available in actual trial. The GL as specified in the original work is used in the evaluation.

#### 5.1.2 Numerical results

Table 3 shows the PCS for I2D, POCRM, the original benchmark (Benchmark), the novel benchmark for partial ordering (PO-Benchmark), and the benchmark by Guo and Liu (2018) (GL). The results of I2D are extracted from Table 2 in the original review, and the results of POCRM are extracted from Table 1 in the comment by Wages (2015).

Comparing the proposed PO-Benchmark and GL approach under scenarios in which the target combination is located on a diagonal (Scenarios 1–3), they provide similar PCS. Under Scenario 4, PO-Benchmark results in 7% higher PCS than the GL approach and performs similar to the original benchmark as there is little uncertainty in the monotonicity associated with the target combination. Under Scenario 5, a similar behavior for the PO-Benchmark is found but the GL approach now corresponds to higher PCS than both the PO-Benchmark and original benchmark that employs the monotonicity assumption.

Under Scenarios 6 and 7, while PO-Benchmark implies that it is more challenging to locate the target combination than, for example, in Scenarios 1–3, the PCS of 65–66% against 73–78%, the GL approach suggests otherwise: PCS of 77–89% against 73–75%. This is counter-intuitive due to fewer target combinations (Scenarios 6) and a more complex interaction mechanism of the compounds (Scenario 7). Under Scenario 7, the GL approach again results in higher PCS than the original benchmark.

Finally, differences between the PO-Benchmark and GL approach can be seen under Scenarios 8–10 with a single target combination. While the PO-Benchmark suggests that these are the most challenging scenarios to find the target combination, the GL suggests that it is, in fact, easier than, for example, Scenario 1 with three target combinations located on the same diagonal. Once more the GL approach, under Scenarios 8 and 9, results in slightly higher or nearly the same PCS as the original benchmark. Consequently, the GL approach does not provide as sharp an upper bound under a number of scenarios, and the PO-Benchmark might provide a more accurate guidance on how challenging each scenarios is under the uncertainty in the ordering.

Under all considered scenarios, the two dose-finding designs result in lower PCS compared to both the original benchmark and the benchmark for partial ordering. Importantly, the original benchmark considered all scenarios with the MTC being not the first or last combination (Scenarios 4 and 5, respectively) as equally difficult with nearly 84% PCS. However, this does not reflect the true challenges that these scenarios impose as they have a different number of the MTCs located at different places on the combination grid. The benchmark for partial ordering recognizes these differences and provides a sharper upper bound for the PCS. Specifically, under Scenario 1, the POCRM and I2D result in 72.8% and 68.0% PCS, respectively. This corresponds to the ratios (with respect to the PO-Benchmark) of 72.8/73.8 = 98.6% and 68.0/73.8 = 92.1%, respectively. At the same time, under Scenario 6, both POCRM and I2D result in a much lower PCS 59.4% and 37.2%. Looking at these values alone (or using the original benchmark) can result in the conclusion that these designs perform poorer in this case compared to Scenario 1. However, the ratio of PCS with respect to the PO-Benchmark is 59.4/65.5 = 90.7% for POCRM and 37.2/65.5 = 56.8% for I2D. Therefore, POCRM still corresponds to a relatively accurate performance, while the I2D design does have potential problems under these scenarios but not as severe as one might conclude by considering the PCS alone.

Regarding the overall performance, POCRM corresponds to a ratio of PCS (compared to PO-Benchmark) of at least 88% under 8 out of 10 scenarios. Under the other two scenarios, Scenario 5 and Scenario 7, the ratio is around 75% which is still relatively high. While further calibration of the model parameters can result in less diverse values of ratios, this is an indication that the POCRM design under the proposed specification is properly calibrated and results in accurate selections under many different scenarios. The I2D design results in the ratio above 87% in 6 out of 10 scenarios. For scenarios 6–7 and 9–10, the I2D design corresponds to ratios of 56.8%, 63.8%, 8.9%, and 23.9%, respectively. This implies that further tuning of the I2D design is required before the design can be applied to an actual clinical trial.

Overall, the novel benchmark has provided noticeable added value over the original benchmark. It leads to the conclusion that the POCRM design results in a good performance in many different scenarios while I2D requires further attention. We refer the reader to Supplementary material available at *Biostatistics* online for another example of the POCRM evaluation with three doses of each drug.

### 5.2 Evaluation of Phase I/II Design for Binary Toxicity and Continuous Efficacy

Below, we evaluate the Phase I/II design for combination trials with binary toxicity and continuous efficacy endpoints proposed by Hirakawa (2012). We refer the reader to Supplementary material available at *Biostatistics* online for the evaluation of Phase I/II design for binary endpoints.

Hirakawa (2012) considered Phase I/II cervical carcinoma trial, in which the squamous cell carcinoma antigen (SCCA) was used as a marker of effect on a continuous scale. Among others, a combination setting with two compounds (*A* and *B*) was considered. There were two doses of drug *A* and four doses of drug *B*. The efficacy outcome was “change in log-transformed SCCA levels from baseline and end of treatment.” Consequently, the lower values of the efficacy outcomes correspond to better performance. It was assumed that the efficacy endpoints has a normal distribution 𝒩(*μ_kl_*, 1) at combination *d_kl_*. The toxicity was evaluated as a binary endpoint characterized by the probability *p_kl_* at combination *d_kl_*. The goal of the combination trial was to find the TC defined as the safe and efficacious combination having the highest efficacy. The upper toxicity bound is *ϕ* = 0.3, and the upper efficacy bound is *ψ* = 0 corresponding to no changes in SCCA levels. To find the target combination, Hirakawa (2012) proposed a model-based approach with a four-parameter combination-toxicity model and an Emax-type seven-parameter combination-efficacy model. The combination selection was based on a Mahalanobis-type distance representing the trade-off between toxicity and efficacy and computed using the posterior distribution of the parameters. We will adopt the notation “Emax” for this design.

The proposed benchmark requires all feasible orderings to be specified. Assuming that the toxicity and efficacy increases with the dose, there are 14 feasible orderings (see Supplementary material available at *Biostatistics* online). Then, the benchmark as in Algorithm 2 with weight function (3.6) and with the binomial likelihood for the toxicity endpoints and the normal likelihood for the efficacy endpoint, assuming that all the orderings are equally likely a priori, can be applied. The following decision criterion is used by Hirakawa (2012)

(5.9) $$R\left(y_{1,kl},y_{2,kl}\right)=\frac{\sum_{i=i}^{n}y_{2,kl}^{\left(i\right)}}{n}\times I\left(\int_{0}^{+\infty }g_{2,kl}\left(v|y_{2,kl}\right)\text{d}v<\eta_{2}\right)\times I\left(\int_{0.3}^{1}g_{1,kl}\left(v|y_{1,kl}\right)\text{d}v<\eta_{1}\right).$$

Three scenarios considered in the original work are given in Table 4, and proportions of each combination selections by the Emax design and respective benchmarks are given in Table 5.

Under Scenario 1, the original benchmark selects *d*_22_ in almost all trials due to the known ordering of toxicities and efficacies. At the same time, the benchmark for partial ordering selects the target combination in 88% of trials with *d*_21_ having the second largest proportion of selections. It also selects *d*_12_ and *d*_13_ with small probabilities. This is in fact in line with the proportion of selections by the Emax design. The ratio of PCS with respect to the PO-Benchmark is nearly 80% against approximately 70% using the original benchmark. Under Scenario 2, both benchmarks lead to the same evaluation of the design resulting in the conclusion that the unknown ordering does not cause any additional obstacles for a design to select the target combination. The ratio of PCS is again nearly 80%. Under Scenario 3, the original benchmark recommends *d*_12_ and *d*_22_ in almost 99% of trials, and never selects *d*_21_ and *d*_31_ as the complete ordering is known. The PO-Benchmark, however, shows that the unknown ordering makes a correct selection more challenging, and selects corresponding suboptimal combinations in 6.5% of trials. This, again, is in line with the Emax design which selects the TC in 44.9% of trials (against 46.8% for the PO-Benchmark—the ratio of PCS is 95%) and combinations *d*_21_ and *d*_13_ in 7.1% of trials.

Overall, the evaluation of the Emax design using the novel benchmark provides the conclusion that the design has high accuracy in all three considered scenarios with the ratio of PCS being above 80%. At the same time, the original benchmark would reveal some problems with the design under Scenario 1, while the performance is as good as under Scenario 2.

## 6 Discussion

A novel benchmark for dose-finding studies with unknown ordering is proposed. The novel benchmark is a generalization of the original proposal by O’Quigley and others (2002) for the setting with unknown ordering. The distinguishing feature of the proposal is that it assesses the complexity of scenarios taking into account not only the uncertainty about the parameters but also the uncertainty about the ordering of these parameters. The proposed benchmark computes the proportions of each combination selection for a given scenario (that might have several combinations with either the same or close toxicity probabilities). It is found that the novel benchmark can provide a more accurate evaluation of dose-finding designs for combination studies than the analysis compared to the original benchmark. The novel approach is easy to implement and does not require any additional information other than that which is available in a simulation study. Finally, the proposed benchmark is computationally feasible even under a large number of orderings as obtaining the benchmark under each ordering has low computational costs.

The proposed benchmark does not select a correct ordering, but in line with the main objective of many Phase I trials, selects the MTC. Moreover, the probability of the ordering being identified as a correct one in itself is not necessarily a useful measure of a good procedure for the MTC selection objective as there may exist multiple orderings that are identical up to the point of the MTC. Either one of these orderings can result in recommending a correct MTC. Consequently, the probability of each ordering is used to compute the probability of the selection under this ordering rather than to select the single ordering and make the inference solely based on it.

Similarly to the original benchmark, the partial ordering benchmark is an evaluation tool that can be used to comprehensively assess the performance of a design that might be considered for a trial. Being a theoretical tool, the benchmark should be used at the planning stage of the trial. Importantly, for the fair and meaningful comparison, the benchmark should use the same criterion for the combination selection as the design under evaluation. The benchmark can also stimulate discussions about the sample size (Cheung, 2013). If in some scenarios, one observes a low PCS under the benchmark, this might indicate that the change in the sample size/number of doses should be explored. At the same time, low PCS should not be interpreted outside of the context as the benchmark accounts for the difficulty of the scenarios. The clinical plausibility of each scenario should be accounted for when interpreting the benchmark’ results. An investigation of the link between the sample sizes and the benchmark performance is subject to future research.

Exploring the behavior of the designs under various assumptions on the correlation between these endpoints and interaction between the compounds might be of interest at the planning stage. The benchmark includes the correlation in its assessment through the algorithm to generate the complete information using the prespecified value of the correlation coefficient. Similarly, the interaction is accounted for implicitly via the simulation scenarios themselves by specifying the toxicity probabilities. In this sense, the proposed benchmark is universal as allows for the assessment of each of these aspects.

While our examples of the benchmark concerned the setting where each of the orderings is equally likely a priori, the benchmark construction allows for prior information about each ordering to be incorporated. As the number of complete orderings can be large, we propose to include this information through the prior information of each combination location in the complete ordering. For example, eliciting the information about the second combination in the complete ordering can be phrased as “What is the probability that the second-lowest dose is *d*_12_?.”

The original benchmark provides an upper bound for the proportion of correct selections as it employs the complete information about each patient. However, it is known that a particular method can provide a higher PCS than the original benchmark under a given scenario if the prior information used is strong enough (Paoletti and others, 2004). The same applies to the benchmark for the partial ordering. Additionally, the proposed benchmark depends on the choice of weight function, *w_kl_*. Whilst we have found that the proposed weight function results in an accurate upper bound for a dose-finding method’s performance in many scenarios, it is possible that the PCS of the evaluated method is greater than the benchmark due to the choice of weight function. Nevertheless, the benchmark still provides a basis for standardization of the PCS that cannot be achieved if analyzing PCS alone—if the ratio of PCSs (compared to the proposed benchmark) is noticeably higher under one scenario than under others, it implies that the design as specified favors the selection of the target combinations under this scenarios.

Finally, it is important to mention that while the proposed benchmark is a useful tool for assessing the performance of any given dose-finding method for combination studies, similar to the benchmark for single-agent studies, it does not capture all aspects of the evaluation. For instance, it does not provide information on the distribution of dose allocation or the average number of DLTs. Developments in these directions are of great value for a more comprehensive assessment of dose-finding designs.

## 7 Software

Software in the form of R code is available on GitHub (https://github.com/dose-finding/combo-benchmark).

## Supplementary Material

Supplementary material is available at is http://biostatistics.oxfordjournals.org.

Supplementary file

## Figures and Tables

**Table 1:** Toxicity probabilities at each combination and corresponding proportions (in %) of each combination selection by the original benchmark based on 10^4^ replications under two combinationtoxicity scenarios with nine drug combinations. The target toxicity level and the selection of the target combinations are in bold.

Toxicity probability									
		Drug B					Drug B		
Scenario 1		*b*_1_	*b*_2_	*b*_3_	Scenario 2		*b*_1_	*b*_2_	*b*_3_
	*a*_1_	0.15	**0.30**	0.45		*a*_1_	0.05	0.15	**0.30**
Drug A	*a*_2_	**0.30**	0.45	0.55	Drug A	*a*_2_	0.15	**0.30**	0.45
	*a*_3_	0.55	0.60	0.65		*a*_3_	0.45	0.55	0.60
Selection proportions									
		Drug B					Drug B		
Scenario 1		*b*1	*b*_2_	*b*_3_	Scenario 2		*b*1	*b*_2_	*b*_3_
	*a*_1_	12.0	**36.5**	7.3		*a*_1_	0.0	5.9	**36.2**
Drug A	*a*_2_	**36.3**	7.3	0.2	Drug A	*a*_2_	6.0	**36.7**	7.2
	*a*_3_	0.3	0.0	0.0		*a*_3_	7.4	0.5	0.0

**Table 2:** Ten considered combination-toxicity scenarios. The MTC is in bold.

Drug A	Drug B					Drug B				
	*b*_1_	*b*_2_	*b*_3_	*b*_4_	*b*_5_	*b*_1_	*b*_2_	*b*_3_	*b*_4_	*b*_5_
	Scenario 1					Scenario 2				
*a*_1_	0.05	0.10	0.15	**0.30**	0.45	0.15	**0.30**	0.45	0.50	0.60
*a*_2_	0.10	0.15	**0.30**	0.45	0.55	**0.30**	0.45	0.50	0.60	0.75
*a*_3_	0.15	**0.30**	0.45	0.50	0.60	0.45	0.55	0.60	0.60	0.80
	Scenario 3					Scenario 4				
*a*_1_	0.02	0.07	0.10	0.15	**0.30**	**0.30**	0.45	0.60	0.70	0.80
*a*_2_	0.07	0.10	0.15	**0.30**	0.45	0.45	0.55	0.65	0.75	0.85
*a*_3_	0.10	0.15	**0.30**	0.45	0.55	0.50	0.60	0.70	0.80	0.90
	Scenario 5					Scenario 6				
*a*_1_	0.01	0.02	0.08	0.10	0.11	0.05	0.08	0.10	0.13	0.15
*a*_2_	0.03	0.05	0.10	0.13	0.15	0.09	0.12	0.15	**0.30**	0.45
*a*_3_	0.07	0.09	0.12	0.15	**0.30**	0.15	**0.30**	0.45	0.50	0.60
	Scenario 7					Scenario 8				
*a*_1_	0.07	0.10	0.12	0.15	**0.30**	0.02	0.10	0.15	0.50	0.60
*a*_2_	0.15	**0.30**	0.45	0.52	0.60	0.05	0.12	**0.30**	0.55	0.70
*a*_3_	**0.30**	0.50	0.60	0.65	0.75	0.08	0.15	0.45	0.60	0.80
	Scenario 9					Scenario 10				
*a*_1_	0.005	0.01	0.02	0.04	0.07	0.05	0.10	0.15	**0.30**	0.45
*a*_2_	0.02	0.05	0.08	0.12	0.15	0.45	0.50	0.60	0.65	0.70
*a*_3_	0.15	**0.30**	0.45	0.55	0.65	0.70	0.75	0.80	0.85	0.90

**Table 3:** Comparison of POCRM and I2D against the benchmark for partial ordering, the original benchmark, and the GL benchmark.

Scenario	1	2	3	4	5	6	7	8	9	10
POCRM	72.8	69.2	69.7	81.0	69.6	59.4	50.0	54.6	51.8	54.1
I2D	68.0	73.7	66.9	89.7	83.7	37.2	41.9	50.4	5.1	13.0
Benchmark	84.1	84.0	84.1	91.1	92.3	84.3	84.2	83.1	83.2	83.2
PO-Benchmark	73.8	78.2	75.9	91.1	92.3	65.5	66.3	57.7	56.0	54.4
GL	73.3	75.0	75.1	84.6	94.6	77.7	89.6	83.8	82.2	76.3

**Table 4:** True values of (*p_kl_*, *μ_kl_*) for each combination of two agents, A and B. The TC is in bold.

		*b*_1_	*b*_2_	*b*_3_	*b*_4_
Scenario 1	*a*_1_	(0.01, 0.5)	(0.10,0.0)	(0.40, −1.5)	(0.50, −2.5)
	*a*_2_	(0.05, −1.5)	**(0.15, −2.0)**	(0.45, −3.5)	(0.55, −4.5)
Scenario 2	*a*_1_	(0.01, 0.0)	(0.05, −0.5)	**(0.15, −3.5)**	(0.45, −5.5)
	*a*_2_	(0.45, −1.0)	(0.50, −1.5)	(0.60, −4.5)	(0.90, −6.5)
Scenario 3	*a*_1_	(0.01, 0.0)	**(0.15, −2.0)**	(0.40, −2.0)	(0.50, −2.0)
	*a*_2_	(0.05, 0.0)	(0.20, −2.0)	(0.45, −2.0)	(0.55, −2.0)

**Table 5:** Comparison of the Emax design and the respective benchmark for partial ordering and the original benchmark. The selections of the TC is in bold.

Design		*b*_1_	*b*_2_	*b*_3_	*b*_4_
Scenario 1					
Benchmark	*a*_1_	0.0	0.0	0.0	0.0
	*a*_2_	0.0	**99.9**	0.1	0.0
PO-Benchmark	*a*_1_	0.0	2.2	1.6	0.0
	*a*_2_	8.1	**88.1**	0.0	0.0
Emax	*a*_1_	0.1	5.1	3.4	0.0
	*a*_2_	14.8	**70.1**	4.7	0.0
Scenario 2					
Benchmark	*a*_1_	0.0	0.0	**99.9**	0.1
	*a*_2_	0.0	0.0	0.0	0.0
PO-Benchmark	*a*_1_	0.0	0.0	**99.9**	0.1
	*a*_2_	0.0	0.0	0.0	0.0
Emax	*a*_1_	4.3	11.5	**78.6**	3.1
	*a*_2_	0.0	0.1	0.3	0.0
Scenario 3					
Benchmark	*a*_1_	0.0	**50.1**	1.2	0.0
	*a*_2_	0.0	47.7	0.0	0.0
PO-Benchmark	*a*_1_	0.0	**46.8**	2.3	0.0
	*a*_2_	4.2	47.3	0.0	0.0
Emax	*a*_1_	0.9	**44.9**	2.8	0.0
	*a*_2_	4.3	45.3	1.8	0.0

## References

[R1] Cheung YK (2013). Sample size formulae for the Bayesian continual reassessment method. Clinical Trials.

[R2] Cheung YK (2014). Simple benchmark for complex dose finding studies. Biometrics.

[R3] Guo B, Liu S (2018). Optimal benchmark for evaluating drug-combination dose-finding clinical trials. Statistics in Biosciences.

[R4] Hirakawa A (2012). An adaptive dose-finding approach for correlated bivariate binary and continuous outcomes in phase I oncology trials. Statistics in Medicine.

[R5] Hirakawa A, Wages NA, Sato H, Andmatsui S (2015). A comparative study of adaptive dose-finding designs for phase I oncology trials of combination therapies. Statistics in Medicine.

[R6] Holmes CC, Walker SG (2017). Assigning a value to a power likelihood in a general Bayesian model. Biometrika.

[R7] Mozgunov P, Jaki T (2019). An information theoretic phase I-II design for molecularly targeted agents that does not require an assumption of monotonicity. Journal of the Royal Statistical Society: Series C (Applied Statistics).

[R8] Mozgunov P, Jaki T (2020). An information theoretic approach for selecting arms in clinical trials. Journal of the Royal Statistical Society Series B (Statistical Methodology).

[R9] Mozgunov P, Jaki T, Paoletti X (2020). A benchmark for dose finding studies with continuous outcomes. Biostatistics.

[R10] O’Quigley J, Paoletti X, Maccario J (2002). Non-parametric optimal design in dose finding studies. Biostatistics.

[R11] Paoletti X, O’Quigley J, Maccario J (2004). Design efficiency in dose finding studies. Computational Statistics & Data Analysis.

[R12] Riviere M-K, Dubois F, Zohar S (2015). Competing designs for drug combination in phase I dose-finding clinical trials. Statistics in medicine.

[R13] Wages NA, Conaway MR, O’Quigley J (2011a). Continual reassessment method for partial ordering. Biometrics.

[R14] Wages NA, O’Quigley J, Conaway MR (2014). Phase I design for completely or partially ordered treatment schedules. Statistics in Medicine.

[R15] Wages NA (2015). Comments on competing designs for drug combination in phase I dose-finding clinical trials by MK. Riviere, F. Dubois, S. Zohar. Statistics in Medicine.

[R16] Wages NA, Conaway MR (2013). Specifications of a continual reassessment method design for phase I trials of combined drugs. Pharmaceutical Statistics.

[R17] Wages NA, Conaway MR, O’Quigley J (2011b). Dose-finding design for multi-drug combinations. Clinical Trials.

[R18] Wages NA, Varhegyi N (2013). pocrm: an r-package for phase i trials of combinations of agents. Computer Methods and Programs in Biomedicine.

[R19] Wages NA, Varhegyi N (2017). A web application for evaluating phase I methods using a non-parametric optimal benchmark. Clinical Trials.

[R20] Wang K, Ivanova A (2005). Two-dimensional dose finding in discrete dose space. Biometrics.

[R21] Yuan Y, Nguyen HQ, Thall PF (2016). Bayesian designs for phase I–II clinical trials.

