# Supplementary material for: A benchmark for dose-finding studies with unknown ordering
Source: Biostatistics. Author manuscript; Available in PMC 2022 Jul 20. (PMC9291639; doi:10.1093/biostatistics/kxaa054)
Supplement: Supplementary file [file EMS144018-supplement-Supplementary_file.pdf]

Biostatistics (2020), **0**, 0, *pp.* 1–15
doi:10.1093/biostatistics/output

A Benchmark for Dose Finding Studies with Unknown Ordering. Supplementary Materials

PAVEL MOZGUNOV*, XAVIER PAOLETTI, THOMAS JAKI
Department of Mathematics and Statistics, Lancaster University, Lancaster, UK
Université Versailles St Quentin & INSERM U900 STAMPM, Institut Curie, Paris, France
p.mozgunov@lancaster.ac.uk

A1. Evaluation of the POCRM design in 3-by-3 scenarios

In this section, we will consider one more example how the novel benchmark can be applied to evalute POCRM design, and, specifically, how it can be used to calibrate the parameters of the POCRM.

Consider a dual-agent combination study with three doses of both drugs (nine combinations), $n = 36$ patients and binary toxicity endpoint. The goal of the trial is to identify the MTC corresponding to the target probability of toxicity $\gamma = 0.30$. We consider nine combination-toxicity scenarios corresponding to different locations of the MTC (Table 1).

The core idea of the POCRM is to run several CRM models under different (pre-specified) complete orderings and allocate patients sequentially based on the most likely ordering. The design requires the specification of several parameters.

Firstly, one need to fix a number of ordering to be considered by the POCRM. Following the recommendation by Wages and Conaway (2013), we specify

*To whom correspondence should be addressed.

© The Author 2020. Published by Oxford University Press. All rights reserved. For permissions, please e-mail: journals.permissions@oup.com

Table 1. Nine considered combination-toxicity scenarios. The MTC is in **bold**.

Drug A	Drug B				Drug B				Drug B		
Sc 1	b_1	b_2	b_3	Sc 2	b_1	b_2	b_3	Sc 3	b_1	b_2	b_3
a_1	.05	.10	.15		.10	.20	**.30**		.20	**.30**	.40
a_2	.12	.20	**.30**		.15	**.30**	.40		**.30**	.40	.50
a_3	.17	**.30**	.45		.17	.40	.50		.40	.50	.55
Sc 4				Sc 5				Sc 6			
a_1	.05	.10	.20		.10	.20	**.30**		.05	.10	.20
a_2	.15	**.30**	.40		**.30**	.40	.50		.10	.15	**.30**
a_3	**.30**	.40	.50		.40	.50	.60		**.30**	.40	.50
Sc 7				Sc 8				Sc 9			
a_1	.05	.17	**.30**		.02	.05	.10		**.30**	.40	.50
a_2	.10	.20	**.30**		.05	.10	.15		.40	.50	.60
a_3	.15	.40	.50		.15	.20	**.30**		.50	.60	.70

1. $d_{11} \to d_{21} \to d_{31} \to d_{12} \to d_{22} \to d_{32} \to d_{13} \to d_{23} \to d_{33}$;
2. $d_{11} \to d_{12} \to d_{13} \to d_{21} \to d_{22} \to d_{23} \to d_{31} \to d_{32} \to d_{33}$;
3. $d_{11} \to d_{21} \to d_{12} \to d_{31} \to d_{22} \to d_{13} \to d_{32} \to d_{23} \to d_{33}$;
4. $d_{11} \to d_{12} \to d_{21} \to d_{13} \to d_{22} \to d_{31} \to d_{23} \to d_{32} \to d_{33}$;
5. $d_{11} \to d_{21} \to d_{12} \to d_{13} \to d_{22} \to d_{31} \to d_{32} \to d_{23} \to d_{33}$;
6. $d_{11} \to d_{12} \to d_{21} \to d_{31} \to d_{22} \to d_{13} \to d_{23} \to d_{32} \to d_{33}$.

and consider each of them being equally likely prior to the trial. Values of the skeletons are defined following the default of the R-package `pocrm` (Wages and Varhegyi, 2013) with the equivalent interval 0.05, and the prior MTC being combination d_{22}.

The proposed benchmark uses all possible $S = 42$ orderings to compute the upper bound. The weight, w_{kl}, for combination kl is computed using the number of combinations with which the combination d_{kl} cannot be ordered (see Supplementary Materials for algorithms computing the orderings and weights).

Table 2 shows the operating characteristics of the POCRM against the original benchmark and the novel benchmark for partial ordering. The results are based on $Z = 40000$ trial replications

to allow considering any difference of more than 1% to be significant. The original benchmark is included to assess the added value the novel benchmark provides. For convenience, we will present the scenarios in rows where the first three values correspond to combinations in the first row of the scenario matrix, the second three to the second, and so on.

Comparing the benchmarks, the proposal results in a lower PCS under all scenarios where the MTC being not the first or last combinations. This is the result of the additional complexity of the scenarios arising from the unknown ordering, which is not taken into account of the original benchmark. The smallest difference of 3% is observed under Scenarios 1 and 3, which correspond to the lowest uncertainty in the ordering for the positions of the MTC. Under Scenario 1, the MTCs are the combinations before the last one, while under Scenario 3 the MTCs are the combinations nearest to the first. In contrast, the largest difference between the benchmarks of 7%-8% are under scenario 2, 5 and 7 where at least one of the MTCs is far from the edges of the combination grid. Moreover, the novel benchmark captures that the MTCs at different positions are not equally difficult to find. For example, under Scenario 5, the MTCs $d13$ and d_{21} are identified in 19% and 25% of trials, respectively, against 26% and 26% by the original benchmark. The difference arises as there is more uncertainty about the positions of the first MTC (standing further from the edges of the combination grid) compared to the second one.

Note that PCS under Scenario 8 and Scenario 9 with the MTC being the last and the first combinations, respectively, are the same for both benchmarks. This reflects that fact that there is no uncertainty about the position of the MTC combination. Whatever the ordering is, the first combination is always first and the last combination is always last. Despite the PCS being the same, the selection proportion of other doses are not always similar. Overall, these results are consistent with the intuition given in the introduction, and the proposed benchmark matches the difficulty of scenarios arising from the unknown ordering.

Considering the benchmark against POCRM, the proposed benchmark provides a tighter

Table 2. Comparison of the POCRM against the benchmark for partial ordering (PO-Benchmark) and the original benchmark. The columns PCS and R correspond to the proportion of correct selections and the ratio of the PCS with respect to the PO-Benchmark. Selection proportions corresponding to the MTC are in bold, and benchmarks' differences in selections of non MTCs of more than 4% with respect to POCRM are underlined.

	d_{11}	d_{12}	d_{13}	d_{21}	d_{22}	d_{23}	d_{31}	d_{32}	d_{33}	PCS	R
Sc 1	.05	.10	.15	.12	.20	**.30**	.17	**.30**	.45		
Benchmark	.00	.00	.01	.00	.17	**.31**	.05	**.31**	.15	.62	
PO-Benchmark	.00	.00	.08	.00	.08	**.29**	.09	**.30**	.15	.59	
POCRM	.00	.01	.09	.01	.12	**.28**	.12	**.27**	.10	.55	93.2%
Sc 2	.10	.20	**.30**	.15	**.30**	.40	.17	.40	.50		
Benchmark	.00	.18	**.26**	.02	**.26**	.11	.04	.11	.02	.52	
PO-Benchmark	.00	.06	**.21**	.03	**.24**	.12	.18	.13	.03	.45	
POCRM	.00	.11	**.18**	.05	**.27**	.11	.14	.14	.01	.45	100%
Sc 3	.20	**.30**	.40	**.30**	.40	.50	.40	.50	.55		
Benchmark	.23	**.26**	.08	**.26**	.08	.01	.08	.01	.00	.52	
PO-Benchmark	.23	**.25**	.09	**.24**	.08	.02	.08	.02	.00	.49	
POCRM	.14	**.24**	.12	**.25**	.10	.02	.12	.02	.00	.49	100%
Sc 4	.05	.10	.20	.15	**.30**	.40	**.30**	.40	.50		
Benchmark	.00	.00	.20	.03	**.26**	.11	**.26**	.11	.02	.52	
PO-Benchmark	.00	.02	.19	.05	**.25**	.13	**.22**	.12	.03	.47	
POCRM	.00	.02	.16	.06	**.28**	.15	**.19**	.12	.02	.47	100%
Sc 5	.10	.20	**.30**	**.30**	.40	.50	.40	.50	.60		
Benchmark	.01	.23	**.26**	**.26**	.11	.01	.11	.01	.00	.52	
PO-Benchmark	.01	.23	**.19**	**.25**	.15	.02	.13	.02	.00	.44	
POCRM	.01	.16	**.24**	**.25**	.17	.03	.12	.02	.00	.49	111.4%
Sc 6	.05	.10	.20	.10	.15	**.30**	**.30**	.40	.50		
Benchmark	.00	.00	.20	.00	.03	**.26**	**.26**	.23	.02	.52	
PO-Benchmark	.00	.00	.14	.00	.14	**.26**	**.21**	.22	.03	.47	
POCRM	.00	.01	.12	.01	.13	**.29**	**.23**	.18	.04	.52	110.6%
Sc 7	.05	.17	**.30**	.10	.20	**.30**	.15	.40	.50		
Benchmark	.00	.05	**.26**	.00	.17	**.26**	.02	.23	.02	.52	
PO-Benchmark	.00	.02	**.19**	.01	.15	**.25**	.12	.24	.03	.44	
POCRM	.00	.04	**.18**	.01	.19	**.21**	.11	.23	.03	.39	88.6%
Sc 8	.02	.05	.10	.05	.10	.15	.15	.20	**.30**		
Benchmark	.00	.00	.00	.00	.00	.02	.02	.21	**.76**	.76	
PO-Benchmark	.00	.00	.01	.00	.00	.09	.01	.13	**.76**	.76	
POCRM	.00	.00	.02	.00	.02	.13	.05	.18	**.60**	.60	78.9%
Sc 9	**.30**	.40	.50	.40	.50	.60	.50	.60	.70		
Benchmark	**.74**	.12	.01	.12	.01	.00	.01	.00	.00	.74	
PO-Benchmark	**.74**	.11	.02	.11	.01	.00	.02	.00	.00	.74	
POCRM	**.57**	.17	.03	.17	.02	.00	.03	.00	.00	.57	77.0%

upper bound for the PCS under scenarios 1-4 and 7-9 than the original one. In these scenarios, the ratio of the PCS is between 77% and 100% which corresponds to a good overall performance

of the dose finding design. However, POCRM results in the ratio of PCS noticeably higher than 100% under Scenario 5 and Scenario 6. This might not be evidence of super-efficiency (Paoletti *and others*, 2004) of POCRM due to the choice of the weight function. The fact that the ratio varies between 77-79% and 110-111% depending on scenario can be a sign that the design is calibrated such that it favours the combination away from the edges of the combination grid. This results in a relatively low ratio of PCS under Scenario 8 with the MTC being the last combination and under Scenario 9 with the MTC being the first combination.

The novel benchmark not only provides a tighter bound but also captures the behaviour of the POCRM in selecting non-MTCs. For example, under Scenario 1, combination d_{13} is selected in 9% of the trials by POCRM and only in 1% by the original benchmark. At the same time, the novel proposal recommends d_{13} in 8% of trials that mimics the behaviour of the POCRM. Similarly, the novel benchmark reflects more accurately the proportion of the combination d_{31} than the original benchmark. Similar patterns can be also found under scenarios 2, 5, 6, 7 and 8. Specifically, under Scenario 8 in which both benchmarks results in the same PCS, POCRM selected d_{23} in 13% of simulated trials against 2% by the original benchmark. The proposal results in a closer proportion of this combination - 9%.

Overall, the novel benchmark allows a more meaningful scenario-specific evaluation of the model-based dose finding design and reveals the uneven performance under various scenarios. Given the evaluation of the benchmark, one can conclude that a further calibration of the POCRM is needed. The example of a benchmark-oriented calibration is given below.

A2. Calibration of POCRM Based on the Novel Benchmark

Given that the maximum likelihood version of the POCRM design is considered, there are two primary design specification that can influence the proportion of correct selections: the chosen orderings and the skeleton. Fixing the skeleton and considering all $S = 42$ orderings in the

POCRM, we find that the PCS is nearly unchanged and the relatively low ratio of PCS (with respect to the novel benchmark) under Scenario 8 and Scenario 9 still remains. Therefore, we focus on the choice of the skeleton below.

The skeleton was obtained using the `getprior` function of the `pocrm` R-package. There are two parameters to be chosen by an investigator: the half-width of the equivalent interval and the location of the prior MTC. The half-width of the equivalent interval was fixed to be .05 and the prior MTC is d_{22}. Below, we investigate how different values of the half-length influence the PCS with respect to the novel benchmark. Specifically, we consider half-lengths of .05, .04 and .03 fixing all of the rest parameters as above.

Table 3 shows the ratio of the PCS for POCRM using the values of half-width with respect to the proposed benchmark. As we evaluate the design over many different scenarios, we will also consider the geometric mean of the ratios to find the design leading to the highest mean ratio over the scenarios (Wages, 2015).

Table 3. Comparison of the POCRM using different values of the half-width (.05,.04,.03) against the benchmark for partial ordering.

Scenario	1	2	3	4	5	6	7	8	9	Mean
POCRM(.05)	93.2	100.0	100.0	100.0	111.4	110.6	88.6	78.9	77.0	94.8
POCRM(.04)	96.6	95.6	98.0	97.9	109.1	108.5	88.6	88.2	83.8	95.9
POCRM(.03)	94.9	93.3	93.9	91.5	104.6	106.4	89.9	96.2	83.9	94.8

The POCRM design using a prior based on a half-width of 0.04 increases the mean ratio by more than 1% compared to the design using a value of 0.05. While the difference might seem marginal, this leads to a more even accuracy across scenarios - the minimum value of the ratio is 83.8% versus 77% using the .05 half-width. A noticeable increase by approximately 10% is observed in the ratio under Scenario 8, while increases of 6.8% and 3.4% is found under Scenarios 10 and 1 respectively. The cost for this improvement is the minor decrease (2%-4.4%) in the ratios in scenarios with the performance close to the benchmark or above benchmark (scenarios 2-6). Using an even smaller value of the half-width of 0.03 results in a reduction of the mean

ratio − 94.8 against 95.9 for the half-width of .04. Therefore, one can recommend the POCRM design using 0.04 for further investigation as it leads to a uniform high accuracy of the MTC selection. Finally, while the mean ratio for a half-width of .05 and .03 are the same, the latter value leads to a more even performance across the scenarios with the minimum ratio being 77% for POCRM(0.05) versus 83.9% for POCRM(0.03). This might make the choice of 0.03 more preferable over 0.05.

B1. Evaluation of the POCRM for Binary Toxicity and Binary Efficacy Endpoints

Wages and Conaway (2014) proposed an extension of the POCRM design for Phase I/II combination studies evaluating toxicity and efficacy simultaneously. The general principle of the POCRM design remains: one needs to run several CRM model for both toxicity and efficacy probabilities (separately) and select the next combination based on the most possible ordering. In the original work, Wages and Conaway (2014) evaluated the POCRM design in the setting of a melanoma clinical trial investigating three doses of one agent (drug A) and two doses of another agent (drug B) that was originally studied by Yin and Yuan (2009). The design was found to outperform the design by Yin and Yuan (2009), in the majority of scenarios. Below, we evaluate the Phase I/II POCRM design in the same setting using the proposed benchmark for two binary endpoints.

The clinical trial investigated two doses of agent A and three doses of agent B. The total sample sizes was fixed to be $n = 80$. Yin and Yuan (2009), and subsequently Wages and Conaway (2014), evaluated their proposals using several combination-toxicity and combination-efficacy scenarios, which are specified in Table 4 with the first number corresponding to the toxicity probability and the second number being the efficacy probability. It assumed that both toxicity and efficacy increase with dose levels of each agent. The goal of the trial was to find the target combination (TC) defined as the efficacious combination ($p_e \geqslant 0.20$) with acceptable toxicity ($p_t \leqslant .30$). Following this definition of the TC, the decision criterion $T(\cdot)$ used by the benchmark to evaluate this design takes the form

$$T(\boldsymbol{y}_{1,kl}, \boldsymbol{y}_{2,jk}) = \mathbb{I}\left(\int_0^{0.2} g_{2,kl}(v|\boldsymbol{y}_{2,kl})\mathrm{d}v < \eta_2\right) \times \mathbb{I}\left(\int_{0.3}^{1} g_{1,kl}(v|\boldsymbol{y}_{1,jk})\mathrm{d}v < \eta_1\right) \tag{0.1}$$

where $g_{1,kl}(\cdot|\boldsymbol{y}_{1,kl})$ and $g_{2,kl}(\cdot|\boldsymbol{y}_{2,kl}$ are probability density functions of a toxicity probability and of an efficacy response given the data, respectively, and η_1, η_2 are threshold probabilities. As the

design considered selects only one TC by the end of the trial, if several combinations satisfy the criterion above, all of them considered as being chosen with equal probability.

Given the monotonicity assumption within each agent, there are five feasible orderings for both toxicity and efficacy. Then, the benchmark uses these five orderings, the decision criterion (0.1) and the weight function (6) as in Algorithm 2 to obtain the proportion of each combination selections. Following Wages and Conaway (2014), the values of probability thresholds $\eta_1 = \eta_2 = 0.95$ were used.

Table 4 shows the operating characteristics of the POCRM as defined by Wages and Conaway (2014) under the specification denoted as (A) in the original work. The results are based on $Z = 10000$ trial replications for the benchmark and 1000 for the design. The results of POCRM are extracted from Supplementary Materials to the original work. The original benchmark is included to assess the added value the novel benchmark provides. Again, the scenarios are presented in rows where the first three values correspond to combinations in the first row of the scenario matrix and the second three to the second row.

Comparing the proposed and original benchmark, noticeable differences in the proportion of correct selections can be found in scenarios 1, 5 and 6. The largest difference is under Scenario 5 with 75% of PCS for the original benchmark and 57% for partial ordering benchmark. This huge differences arises from that fact that the original benchmark never selects combination d_{21} due to the known monotonic ordering: given the complete information d_{21} has nearly the same toxicity but much lower efficacy and should be never selected under the assumption of known ordering. In fact, there is uncertainty between the target combination d_{12} and d_{21}. The partial ordering benchmark is able to capture this uncertainty and provide a more meaningful tool for the comparison. While the PCS under other three scenarios are not different, the proportions of non-target combination differ, providing an insight on the distribution of selection under the complete information.

Table 4. Comparison of the POCRM design against the benchmark (B.) and the benchmarkfor partial ordering (PO-B.). Selection proportions corresponding to the acceptable (safe and efficacious) combinations are in bold. The columns R_1 and R_2 correspond to the ratios of the PCS of the POCRM compared to the B. and PO-B., respectively. The selection proportions of a suboptimal combinations that are tighter bounded by the PO-Benchmark than by the Benchmark are underlined.

	d_{11}	d_{12}	d_{13}	d_{21}	d_{22}	d_{23}	R_1	R_2
Sc 1	(.05,.10)	(.15,.30)	(.20,.50)	(.10,.20)	(.15,.40)	(.45,.60)		
B.	.00	.00	**.89**	.00	.00	.11		
PO-B.	.00	.00	**.80**	.00	.09	.11		
POCRM	.00	.01	**.56**	.00	.22	.21	62.9	70.0
Sc 2	(.05,.20)	(.20,.40)	(.50,.55)	(.10,.30)	(.40,.50)	(.60,.60)		
B.	.00	**.63**	.01	.00	.36	.00		
PO-B.	.00	**.63**	.05	.01	.31	.00		
POCRM	.00	**.43**	.10	.15	.32	.00	68.2	68.2
Sc 3	(.05,.10)	(.15,.30)	(.20,.40)	(.10,.20)	(.20,.40)	(.50,.50)		
B.	.00	.00	**.50**	.00	**.50**	.00		
PO-B.	.00	.00	**.49**	.00	**.49**	.02		
POCRM	.00	.00	**.42**	.00	**.45**	.12	87.0	88.7
Sc 4	(.23,.36)	(.40,.49)	(.59,.62)	(.40,.44)	(.72,.58)	(.90,.71)		
B.	**.62**	.37	.00	.00	.00	.00		
PO-B.	**.62**	.22	.00	.16	.00	.00		
POCRM	**.55**	.25	.00	.19	.00	.00	88.7	88.7
Sc 5	(.13,.32)	(.25,.50)	(.42,.68)	(.24,.40)	(.56,.60)	(.83,.78)		
B.	.00	**.75**	.25	.00	.00	.00		
PO-B.	.00	**.57**	.21	.18	.03	.00		
POCRM	.01	**.61**	.10	.27	.02	.00	81.3	107.0
Sc 6	(.11,.15)	(.15,.22)	(.20,.31)	(.15,.30)	(.25,.41)	(.40,.54)		
B.	.00	.00	.00	.00	**.62**	.38		
PO-B.	.00	.00	.08	.00	**.55**	.37		
POCRM	.00	.01	.21	.09	**.52**	.16	83.9	94.5

Comparing the POCRM design to the benchmark for partial ordering, POCRM results in quite different ratios of the PCS under different scenarios. Under scenario 1 and 2, the ratio is around 70%, while the ratio under scenarios 3, 4 and 6 varies between 88%-95%. Interestingly, the ratio reaches 107% under Scenario 5. The variation of the ratio of selections across scenarios might be a sign that further calibration of the design might be necessary to achieve a more uniform performance over the considered scenarios. Importantly, the proposed benchmark result in a different conclusion on the assessment of the design compared to the use of the original benchmark. Indeed, using the original benchmark, one can conclude that the design performs

less accurately under Scenario 5 than under scenarios 3, 4 and 6. However, using the benchmark for partial ordering, the design leads to a higher ratio of PCSs. Furthermore, the partial ordering benchmark reveals that the POCRM design performance is not as low as the original benchmark proposes under scenarios 1 and 6.

Overall, the partial ordering benchmark matches the distribution of selections of the POCRM design more accurately than the original benchmark, and provides a more meaningful evaluation of the dose finding design under the majority of scenarios.

Orderings

The proposed benchmark requires the specification of all feasible orderings. Below, we provide the list of these orderings in scenarios with low and moderate number of doses. For the setting with 3 and 5 doses of each compound (and 6006 feasbile orderings), we provide a computationally cheap algorithm to obtain them. In both cases, the codes to compute orderings are available on GitHub (`https://github.com/dose-finding/combo-benchmark`). For the sake of simplicity, we will adopt the convention that that the combination of d_{11} is called the first combination and is coded as "1" and we then number all combinations consecutively by rows. For example, d_{12} is coded as "2", d_{13} as "3" and so on.

C1. Orderings

Table 5. All feasible orderings in dual-agent trial with 2 doses of agent A and 4 doses of agent B used to evalute the design by Hirakawa (2012).

Ordering								
1	1	2	3	4	5	6	7	8
2	1	2	3	5	4	6	7	8
3	1	2	5	3	4	6	7	8
4	1	5	2	3	4	6	7	8
5	1	2	3	5	6	4	7	8
6	1	2	5	3	6	4	7	8
7	1	5	2	3	6	4	7	8
8	1	2	5	6	3	4	7	8
9	1	5	2	6	3	4	7	8
10	1	2	3	5	6	7	4	8
11	1	2	5	3	6	7	4	8
12	1	5	2	3	6	7	4	8
13	1	2	5	6	3	7	4	8
14	1	5	2	6	3	7	4	8

Table 6. All feasible orderings in a dual-agent trial with 3 doses of each compound used to evalute the POCRM design in A1.

Ordering									
1	1	2	3	4	5	6	7	8	9
2	1	2	4	3	5	6	7	8	9
3	1	4	2	3	5	6	7	8	9
4	1	2	4	5	3	6	7	8	9
5	1	4	2	5	3	6	7	8	9
6	1	2	3	4	5	7	6	8	9
7	1	2	4	3	5	7	6	8	9
8	1	4	2	3	5	7	6	8	9
9	1	2	4	5	3	7	6	8	9
10	1	4	2	5	3	7	6	8	9
11	1	2	3	4	7	5	6	8	9
12	1	2	4	3	7	5	6	8	9
13	1	4	2	3	7	5	6	8	9
14	1	2	4	5	7	3	6	8	9
15	1	4	2	5	7	3	6	8	9
16	1	2	4	7	3	5	6	8	9
17	1	4	2	7	3	5	6	8	9
18	1	2	4	7	5	3	6	8	9
19	1	4	2	7	5	3	6	8	9
20	1	4	7	2	3	5	6	8	9
21	1	4	7	2	5	3	6	8	9
22	1	2	3	4	5	7	8	6	9
23	1	2	4	3	5	7	8	6	9
24	1	4	2	3	5	7	8	6	9
25	1	2	4	5	3	7	8	6	9
26	1	4	2	5	3	7	8	6	9
27	1	2	3	4	7	5	8	6	9
28	1	2	4	3	7	5	8	6	9
29	1	4	2	3	7	5	8	6	9
30	1	2	4	5	7	3	8	6	9
31	1	4	2	5	7	3	8	6	9
32	1	2	4	7	3	5	8	6	9
33	1	4	2	7	3	5	8	6	9
34	1	2	4	7	5	3	8	6	9
35	1	4	2	7	5	3	8	6	9
36	1	4	7	2	3	5	8	6	9
37	1	4	7	2	5	3	8	6	9
38	1	2	4	5	7	8	3	6	9
39	1	4	2	5	7	8	3	6	9
40	1	2	4	7	5	8	3	6	9
41	1	4	2	7	5	8	3	6	9
42	1	4	7	2	5	8	3	6	9

C2. An Algorithm for Computing Approximate Number of Orderings in Trials with Many Doses

For a large number of combinations, the computation exact number of orderings can be computationally expensive. Therefore, we propose the following simulation-based approach to find the orderings. Essentially, the procedure builds on the idea by Clertant and O'Quigley (2017)

Table 7. All feasible orderings in dual-agent trial with 2 doses of agent A and 3 doses of agent B used to evalute the POCRM design for Phase I/II clinical trial in B1.

Ordering						
1	1	2	3	4	5	6
2	1	2	4	3	5	6
3	1	4	2	3	5	6
4	1	2	4	5	3	6
5	1	4	2	5	3	6

to randomly simulate scenarios satisfying the monotonicity assumption within each agent. Then, for a large enough number of simulated scenarios unique orderings can be identified, which will be the orderings used by the benchmark.

Let us consider the example computing the ordering in the setting with 3 and 5 doses of agents used to evalute the POCRM and I2D designs in the main body of the manuscript. Fixing the value of parameter at the first and last combinations as $p_{11} = 0$ and $p_{35} = 1$, one can draw a probability of toxicity at combination d_{21} as $p_{21} \sim \mathcal{U}(p_{11}, p_{35})$. Then, the probability of toxicity at combination d_{31} should be drawn from $p_{31} \sim \mathcal{U}(p_{21}, p_{35})$, and so on. The bounds of the uniform distribution are chosen such that the obtained scenario satisfies the monotonicity assumption within each agent. The procedure starts from the combination d_{11} and proceeds by rows. For example, the toxicity probability at combination d_{23} is generated as $p_{23} \sim \mathcal{U}(min(p_{13}, p_{22}), p_{35})$. The algorithm proceeds until all the probabilities for all 15 combinations are obtained. Using $Z = 10^6$ simulations (an average computational time is 45 sec), one can obtain that there are 6006 feasible orderings satisfying the monotonicity assumption within each agent. Note that the same approach can be use for studies with non-binary endpoints.

References

Clertant, M. and O'Quigley, J. (2017). Semiparametric dose finding methods. *Journal of the Royal Statistical Society: Series B (Statistical Methodology)* **79**, 1487–1508.

Paoletti, X., O'Quigley, J. and Maccario, J. (2004). Design efficiency in dose finding studies. *Computational Statistics & Data Analysis* **45**, 197–214.

Wages, N.A. and Conaway, J. O'Quigleyand M. R. (2014). Phase I design for completely or partially ordered treatment schedules. *Statistics in Medicine* **33**, 569–579.

Wages, N. A. (2015). Comments on 'competing designs for drug combination in phase I dose-finding clinical trials' by MK. Riviere, F. Dubois, S. Zohar. *Statistics in Medicine* **34**, 18.

Wages, N. A. and Conaway, M. R. (2013). Specifications of a continual reassessment method design for phase I trials of combined drugs. *Pharmaceutical Statistics* **12**, 217–224.

Wages, N. A. and Varhegyi, N. (2013). pocrm: An r-package for phase i trials of combinations of agents. *Computer methods and programs in biomedicine* **112**, 211–218.

Yin, G. and Yuan, Y. (2009). Bayesian dose finding in oncology for drug combinations by copula regression. *Journal of the Royal Statistical Society: Series C (Applied Statistics)* **58**, 211–224.

[*Received August 1, 2010; revised October 1, 2010; accepted for publication November 1, 2010*]
